# U-Tube in High Bileduct Obstruction

**DOI:** 10.1155/1995/62578

**Published:** 1995

**Authors:** J. Charles Yeo

**Affiliations:** Department ofSurgery; Blalock 606 The Johns Hopkins Hospital, 600 N. Wolfe Street Baltimore MD 21287-4606 USA


*HPB Surgery*, 1995, Vol. 8, pp. 201–212
Reprints available directly from the publisher
Photocopying permitted by license only

© 1995 Harwood Academic Publishers GmbH
Printed in Malaysia

### HPB INTERNATIONAL

**EDITORIAL & ABSTRACTING SERVICE**

JOHN TERBLANCHE, EDITOR
Department of Surgery
Medical School · Observatory 7125
Cape Town · South Africa
Telephone: (021) 406-6232
Telefax (021) 448-6461

### U-TUBE IN HIGH BILE DUCT OBSTRUCTION

### ABSTRACT

*Millikan, K. W., Gleason, T. G., Deziel, D. J. and Doolas, A. (1993) The current role of U tubes for benign and malignant biliary obstruction. Annals of Surgery; 218, 621–629.*

***Objective.*** **The recent experience with U tubes at Rush-Presbyterian-St. Lukes Medical Center was reviewed in order to assess their current role in hepatobiliary surgery.**
***Summary Background Data.*** **Transhepatic intubation by a variety of methods has been used routinely for biliary decompression and inhibition of anastomotic stricture since the 1960s. U tubes were popularized in the early 1970s. However, little has been written about their use and efficacy in recent years. Because of the apparent benefits associated with the use of U tubes versus other stenting techniques, the authors performed this study.**
***Methods.*** **The hospital and office charts of all patients who had U tubes placed between 1980 and 1992 were reviewed retrospectively. Between 1980 and 1992, U tubes were placed intraoperatively in 54 patients for biliary decompression and/or stenting. Twelve patients were operated on for benign causes of obstruction. Forty-two patients with malignant tumors underwent surgery for U tube placement in conjunction with or without tumor resection and anastomotic bypass.**
***Results.*** **There was a 0% operative mortality rate in the benign group. In six patients, the U tube played a major role in the long-term management of their disease processes. None of these patients has had restricture since removal of the tube. In the malignant group, the 30-day operative mortality rate was 12%. After 3 months, marked clinical improvement and complete biliary decompression were achieved, with mean bilirubin levels dropping from 14.0 mg/dL to 1.3 mg/dL. No patients in the malignant group required reoperation for recurrent biliary obstruction after u tube placement.**
***Conclusions.*** **The use of U tubes is advocated for biliary decompression and/or anastomotic stenting in patients with benign stricture or resectable malignancy and in patients with nonresectable, malignant biliary obstruction for adequate palliation of intractable jaundice.**

KEY WORDS: Biliary obstruction biliary stricture bile duct tumor U-tube stent.

### PAPER DISCUSSION

The paper by Millikan *et al.* is a retrospective review of the use of U tubes for biliary decompression from the Rush-Presbyterian-St. Lukes Medical Center, spanning the years 1980 to 1992. The authors provide detailed data on the use of U tubes in 54 patients, with 12 patients having benign strictures and 42 patients having malignant obstruction. The report collects together patients treated with stricture resection, as well as those treated by palliative bypass (with or without biliary-enteric anastomosis). The authors conclude that the U tube is effective for the treatment of biliary strictures, and that it has advantages over the other types of biliary stents.

The controversy over the best type of operatively-placed transhepatic stent remains unsettled by this report[1,2]. Supporters of U tubes (which exit the skin at two sites) favor their use because of the stated ease of exchange and high levels of patient acceptance with the cleaning procedure. Supporters of straight transhepatic tubes (which exit the skin at only one site, with the internal aspect of the stent typically residing in a Roux-en-Y loop of bowel used for biliary-enteric anastomosis) favor their use because the number of exit sites per patient is reduced and the tube exchange process has become a simple outpatient procedure. Prospective data comparing U tubes and straight tubes are lacking, and thus the resolution of this controversy remains largely emotional and based upon the surgeon's preference. We favor straight transhepatic tubes, which are often inserted using pre-existing percutaneously-placed transhepatic "Ring" catheters, or can be inserted transhepatically at the time of laparotomy[3]. Several issues addressed by the article of Millikan *et al.* deserve further mention: the challenging pattern of iatrogenic bile duct injuries, the emergence of non-surgical options for bile duct obstruction, and the need for a multidisciplinary approach to many patients with obstructive jaundice.

First, in their group of 12 patients with benign bile duct strictures only one stricture involved the common hepatic duct and only one stricture was above the hepatic duct bifurcation. The recent introduction, popularization and widespread use of laparoscopic cholecystectomy has been associated with an increased incidence of bile duct injuries, many of which involve the common hepatic duct or its bifurcation[4–6]. Repair of such bile duct injuries has been reported both early and late after their occurrence, and not uncommonly has required bilateral hepaticojejunostomy, with transhepatic stents used to traverse both the right and left lobes of the liver.

Second, Millikan *et al.* treated 42 patients with malignant biliary obstruction with U tube stenting, with 18 patients undergoing tumor resection (9 "curative", 9 palliative) and 24 patients undergoing U tube placement for palliation, without tumor resection. In this entire group of patients the 30 day postoperative mortality rate was 12% and in-hospital mortality rate was 26%, rates that are similar to some series[7,8] but not much higher than other reported concurrent series[8,9]. One explanation for this high in-hospital mortality rate may involve the choice of surgical U tube palliation over other nonoperative options such as percutaneous or endoscopic palliation. A nonoperative approach to palliation may be undertaken because the tumor is considered unresectable by preoperative staging studies or because the patient is considered unfit for surgery. Patients unfit for surgery include those with poor performance status, distant metastases, extensive tumor extension into both the right and left lobes of the liver, and portal vein or main hepatic artery occlusion.

Percutaneous transhepatic palliation usually involves the ultimate placement of 12 to 16 French soft silastic transhepatic catheters through the tumor and into the duodenum. In most cases these percutaneous stents are exchanged over guidewires as an outpatient procedure every three to four months, to avoid the complications of side hole occlusion, biliary obstruction, and cholangitis. A further percutaneous palliative option is the transhepatic placement of a biliary endoprosthesis. New metallic devices the Wallstent, Gianturco Z-stent, and Palmaz stent. Should the endoscopic route be chosen, plastic biliary endoprostheses varying in diameter from 7 to 11.5 French can be successfully inserted into the obstructed biliary tree. Plastic endoprostheses appear to have a higher occlusion rate than metal endoprostheses, as several randomized controlled studies have now shown that self-expanding metals stents (Wallstent) are superior to 10 French plastic stents for palliation of jaundice in malignant bile duct obstruction. For example, a recent study from Amsterdam randomized 105 patients with distal strictures to metal versus plastic stents[10]. The success of the initial drainage was 95% with both treatments, but the median patency was significantly longer for the metal stent (273 days) as compared to the plastic stent (126 days), with fewer endoscopic reinterventions needed for the metal stent.

The choice of nonoperative versus operative palliation in patients with malignant biliary obstruction remains difficult. Several randomized, controlled studies have compared these different modes of palliation. Bornman *et al.* compared percutaneous stenting with surgery in 50 patients with distal bile duct obstruction[11]. Technical success was achieved in 84% of the patients in the percutaneously stented group, and in 76% of the patients in the surgery group. The procedure-related complications (28% and 32%, respectively) and 30 day mortality (8% and 20%) were similar. Although the initial hospital stay was significantly shorter in the stented group (18 vs 24 days), this difference was not maintained when readmissions for obstructed endoprostheses and duodenal obstruction were also considered. Another study by Dowsett *et al.* included 127 patients with unresectable malignancy obstructing the *distal* bile duct[12]. Sixty-five patients were treated via endoscopic stenting and 62 had surgical palliation. Successful biliary drainage was achieved in 94% of patients, with the 30 day mortality being 6% after endoscopy and 15% after surgery. However, recurrent jaundice (17% vs 3%) and late duodenal obstruction (14% vs 3%) were seen more commonly in the endoprosthesis group. Unfortunately, no prospective randomized studies have compared surgical to nonsurgical palliation of *hilar* cholangiocarcinoma or more proximal biliary obstruction, and therefore data applicable to this situation are lacking.

In conclusion, the management of patients with obstructive jaundice from either benign or malignant processes no longer resides solely within the hands of surgeons. A multidisciplinary approach to such a problems is currently indicated, with some patients being best treated by endoscopic or percutaneous techniques, others by surgical techniques, and still others using a multidisciplinary approach. Operatively placed transhepatic stents continue to play an important role in the management of these patients. So do the procedures performed by our talented endoscopists and interventional radiologists.

*References*

1. Terblanche, J., Kahn, D., Bornman, P. C., Werner, D. (1988) The role of U tube palliative treatment in high bile duct carcinoma. *Surgery*, **103**, 624–632.
2. Cameron, J. L., Broe, P., Zuidema, G. D. (1982) Proximal bile duct tumors. Surgical management with silastic transhepatic biliary stents. *Ann. Surg.*, **196**, 412–419.
3. Yeo, C. J. and Cameron, J. L. (1992) Transhepatic biliary stents in high benign and malignant biliary tract obstructions. In: *Mastery of Surgery*, 2nd edition, Nyhus, L. M. and Baker, R. J. (ed.), Boston, Little Brown and Co., 960–967.
4. Davidoff, A. M., Pappas, T. N., Murray, E. A. *et al.* (1992) Mechanisms of major biliary injury during laparoscopic cholecystectomy. *Ann. Surg.*, **215**, 196–208.
5. Rossi, R. L., Schirmer, W. J., Braasch, J. W. *et al.* (1992) Laparoscopic bile duct injuries. Risk factors, recognition and repair. *Arch. Surg.*, **127**, 596–602.
6. Ress, A. M., Sarr, M. G., Nagorney, D. M. *et al.* (1993) Spectrum and management of major complications of laparoscopic cholecystectomy. *Am. J. Surg.*, **165**, 655–662.
7. Lai, E. C. S., Chu, K. M., Lo, C. Y. *et al.* (1992) Surgery for malignant obstructive jaundice: Analysis of martality. *Surgery*, **112**, 891–896.
8. Ottow, R. T., August, D. A., Sugarbaker, P. H. (1985) Treatment of proximal biliary tract carcinoma: An overview of techniques and results. *Surgery*, **97**, 251–262.
9. Cameron, J. L., Pitt, H. A., Zinner, M. J. *et al.* (1990) Management of proximal cholangiocarcinomas by surgical resection and radiotherapy. *Am. J. Surg.*, **159**, 91–98.
10. Davids, P. H. P., Groen, A. K., Rauws, E. A. J. *et al.* (1992) Randomized trial of self-expanding metal stents versus polyethylene stents for distal malignant biliary obstruction. *Lancet*, **340**, 1488–1492.
11. Bornman, P. C., Harries-Jones, E. P., Tobias, R. *et al.* (1986) Prospective controlled trial of transhepatic biliary endoprosthesis versus bypass surgery for incurable carcinoma of the head of the pancreas. *Lancet*, **1**, 69–71.
12. Dowsett, J. F., Russell, R. C. G., Hatfield, A. R. W. *et al.* (1989) Malignant obstructive jaundice: A prospective randomized trial of bypass surgery versus endoscopic stenting. *Gastroenterology*, **96**, A128.

J. Charles Yeo, M.D.
Department of Surgery; Blalock 606
The Johns Hopkins Hospital
600 N. Wolfe Street
Baltimore, MD 21287-4606
Phone No: (410) 955-7496
FAX No: (410) 614-3539

### LIVER RESECTION UNDER ISCHEMIA: INFLOW OCCLUSION OR TOTAL HEPATIC ISOLATION

### ABSTRACT

*Huguet, C., Gavelli, A., Chieco, P. A., Bona, S., Harb, J., Joseph, J. M., Gramaglia, M. and Lasserre, M. (1992) Liver ischemia for hepatic resection: Where is the limit? Surgery; III: 251–259.*

